# Immobilization and Purification of Enzymes With the Novel Affinity Tag ChBD-AB From *Chitinolyticbacter meiyuanensis* SYBC-H1

**DOI:** 10.3389/fbioe.2020.00579

**Published:** 2020-06-12

**Authors:** Jie Zhou, Jianhao Chen, Nisha Zhuang, Alei Zhang, Kequan Chen, Ning Xu, Fengxue Xin, Wenming Zhang, Weiliang Dong, Min Jiang

**Affiliations:** ^1^State Key Laboratory of Materials-Oriented Chemical Engineering, College of Biotechnology and Pharmaceutical Engineering, Nanjing Tech University, Nanjing, China; ^2^Jiangsu National Synergetic Innovation Center for Advanced Materials, Nanjing Tech University, Nanjing, China

**Keywords:** chitin-binding domain, fusion tag, affinity chromatography, protein purification, protein immobilization, enzyme conversion

## Abstract

A new protein immobilization and purification system has been developed based on the improved plasmid vectors, designated pETChBD-X, which contained the gene coding for two novel chitin-binding domains ChBD-AB, factor Xa cleavage site and adapted for gene fusions. The ChBD-AD from *Chitinolyticbacter meiyuanensis* SYBC-H1 was used as a novel affinity tag to anchor fusion proteins to chitin granules. The granules carrying the ChBD-AD fusion proteins can be isolated by a simple centrifugation step and used directly for some applications. Moreover, when required, a practically pure preparation of the soluble recombination protein can be obtained after Factor Xa cleavage. The efficiency of this system has been demonstrated by reaching 95% of protein absorbed to chitin within 30 min and recycling over 75% of interest protein after Factor Xa cleavage to separate interest protein and fusion tag. Furthermore, 65% L-glutamate oxidase with this fusion tag could be purified and immobilized within only one step and to be reused in converting L-glutamate to α-ketoglutaric acid directly, the average conversion rate kept above 65% even within four batches of enzyme conversion reaction.

## Introduction

Chromatography is an important biological technique used for separating, identifying, and purifying target proteins from a mixture based on different characteristics such as size and shape, total charge, hydrophobic groups present on the surface and so on (Coskun, 2016). Nowadays, a variety of chromatography methods are widely applied in various occasions where protein purification or immobilization are required. Thus, by fusing the coding sequence of the interest protein with different tag sequences, which have high affinity to a ligand, these protein can be efficiently purified for identification and characterization (Li et al., 2017).

Protein affinity tag is an indispensable tool for recombinant protein expression and purification. The invention and application of these fusion tags including C-myc, HA, FLAG, poly-arginine or histidine, streptavidin binding tag, et al., have undoubtedly facilitated the in-depth research and practical application of various proteins (Jin and Amarasinghe, 2015). However, traditional affinity chromatography methods are always high cost and cumbersome operation, thus it appears important to develop new affinity chromatography methods to overcome these shortcomings.

Carbohydrate-binding module (CBM) including ChBD is found extensively in glycoside hydrolases, it is a sort of non-catalytic domain performing carbohydrate-binding activity and promoting the association of the enzyme with the substrate (Boraston et al., 2004). In view of high absorbing capacity to substrate, CBM proteins show potential application in feed industry, biomedicine, environmental protection and molecular biology (Oliveira et al., 2015). Recently, CBMs have been developed as useful affinity tags applied in purification and immobilization of proteins. For example, Rodriguez et al. (2004) used cellulose binding-domain as affinity tag and the purity of target protein reached over 95%; Demishtein et al. (2010) used a modified CBM as affinity tag and finally recovered about 90% of target protein; Liao et al. (2012) reported an example of one-step purification and immobilization of thermophilic polyphosphate glucokinase by using a family 3 cellulose-binding domain as fusion tag; Qin et al. (2019) realized one-step immobilization and purification of enzymes by carbohydrate-binding module family 56 tag fusion. All the examples above concluded that CBMs are practical and reliable fusion tags which have great potential for applications in biology research. When being used as affinity tags, CBMs usually possess two obvious advantages: high specificity and low cost. In general, the ability of binding specific substrates is unique to CBM, this uniqueness can prevent non-specific binding of other protein. In addition, the substrates which can bind CBMs, such as cellulose, chitin, xylan, starch and so on, are usually low-cost because of their widespread in nature.

In this work, we successfully truncated two potential chitin-binding domains (ChBD-AB) of chitinase *Cm*Chi1 from *Chitinolyticbacter meiyuanensis* SYBC-H1 according to amino acid analysis and confirmed its affinity ability to chitin which may be valuable in applying as fusion tag (Zhang et al., 2018). We constructed a novel protein immobilization and purification system with these ChBD-AB, and the universal plasmid vector (pETChBD-X, X represents gene encoding interest protein) contained the gene coding the two novel chitin-binding domains ChBD-AB, factor Xa cleavage site has been improved. The usefulness of these vector were exemplified by fusions to the typical genes encoding green fluorescent protein (GFP) and L-glutamate oxidase (LGOX). The visible purifying process and efficiency of purification and immobilization were evaluated. One-step immobilized L-glutamate oxidase (LGOX) in converting L-glutamate to α-ketoglutaric acid (α-KG) directly were established. This may provide a useful purification and immobilization platform for researching and application of recombinant proteins.

## Materials and Methods

### Reagents and Strains

Chitin powder was purchased from Sinopharm Chemical Reagent, Co. (Beijing, China). Molecular biological reagents including DNA polymerase and restriction enzymes (*Nde* I, *Xho* I) purchased from TaKaRa Biotechnology, Co. (Dalian, China). Exnase required for one step cloning was purchased from Vazyme Biotech, Co. (Nanjing, China). Relevant molecular reagents kits including bacteria genomic DNA kit, plasmid miniprep kit, gel mini purification kit and SDS-PAGE kit were purchased from Zoman Biotechnology, Co. (Beijing, China).

The lysogeny broth (LB) contained 10 g/L tryptone, 5 g/L yeast extract, and 5 g/L NaCl. Expression vector pET-29a were used to fusion target gene fragments while *Escherichia coli* BL21 (DE3) was used as host strain in this work. All strains were cultivated in LB medium.

### Plasmid and DNA Manipulations

The ChBD-AB gene, which was encoding two ChBDs modules of chitinase *Cm*Chi1, was amplified by polymerase chain reaction (PCR) from genome of *Chitinolyticbacter meiyuanensis* SYBC-H1 and a fragment encoding factor Xa cleavage site was added at the primer. The PCR reactions were performed for 32 cycles (95°C for 30 s, 60°C for 30 s, 72°C for 90 s), followed by a 10 min extension at 72°C. Then the amplified PCR products were purified and ligated into the pET29a vector. This way, one universal plasmid vector pETChBD-X was generate (Figure 2).

Then, the gene fragments encoding GFP and LGOX respectively were inserted multi clone site (MCS) by one step cloning for further study. After transformation into *E. coli* DH5α, the positive transformants were screened and verified by DNA sequencing. plasmid pETChBD-GFP and pETChBD-LGOX with a C-terminal His 6-tag were obtained. The recombinant plasmid was transformed into competent *E. coli* BL21(DE3) for protein expression. Sequencing service was supplied by GenScript Biotech Co. (Nanjing, China).

### Overexpression and Purification of Proteins

*Escherichia coli* BL21(DE3) harboring pETChBD-X, pETChBD-GFP, and pETChBD-LGOX were inoculated into LB medium containing kanamycin at final concentration of 50 mg/L and incubated at 37°C with a rotation speed of 180 rpm. After the optical density (OD_600_) of the culture broth reached 0.6–0.8, isopropyl β-D-thiogalactopyranoside (IPTG) was added to a final concentration of 1 mM, and the culture was further grown at 20°C and with a rotation speed of 120 rpm for 24 h.

The bacterial cells were harvested by centrifugation at 2124 × *g* and 4°C for 10 min, washed twice with equilibration buffer (20 mM Tris-HCl buffer, pH 7.0) at 4°C and then disrupted by ultrasonication. After, the cell debris was removed by centrifugation at 12,580 × *g* and 4°C for 20 min, and the supernatant was retained as crude extract. The target recombinant proteins ChBD-AB was purified by Ni-NTA agarose column. Sodium dodecyl sulfate-polyacrylamide gel electrophoresis (SDS-PAGE) was carried out to analyze purification steps of the target proteins using standard methods.

### Preparation of Ultrasonic Wave Chitin Powder and Colliodial Chitin

Ultrasonic wave chitin powder (UWCP) was prepared by ultrasonic crusher previously described. 20 g/L of chitin powder suspension was treated by sonication, the parameters were set to 25 kHZ, 300 W for 20 min. During the whole process, the suspension was cooled with ice water.

The preparation of colloidal chitin (CC) was referred to Inokuma et al. (2013) method. 10 g chitin powder was soaked by 40 mL acetone for 2 min and then been mixed with 100 mL concentrated hydrochloric acid (35%). The mixture were stirred with magnetic stirrer until it became transparency and then pored into 2000 mL cold ethanol (50%). After being fully stirred, the solution was left in the refrigerator overnight at 4°C with 100 mL of 35% hydrochloric acid. The mixture was stirred with magnetic stirrer until it became transparency and then pored into 2000 mL cold ethanol. Repeat the above steps and rinse colloidal chitin for several times until the pH value of the resuspension was around 6.0. Finally, colloidal chitin was prepared at 1% concentration.

### Chitin-Binding Assay

Chitin was added to solution containing purified ChBD-AB protein, then the suspension was incubated under different conditions. After the adsorption was completed, solid was removed by centrifugation. Residual protein concentration in supernate was detected. In this way, the adsorption rates were calculated as follows:

$$\text{Adsorption rate}=\left(1-\frac{\text{Protein quality in supernate}}{\text{Initial protein quality}}\right)\times 100\%.$$

Detection of protein concentration was referred to Bradford method (Campion et al., 2017). Four factors were taken into account to investigate the effects on absorption: material of adsorbent, temperature, adsorbent concentration and incubating time. Four principal factors are systematically examined using orthogonal design of experiments (L9 matrix) based on the results of single-factor experiments, different experiments were designed according to different conditions and factors were independent and non-interference. Statistical analysis was used to identify the order of principal factors in terms of adsorption rates. Results including optimal condition and impact level of different factors were analyzed using MINITAB 14 software (Wu and Leung, 2011).

With regards to ChBD-GFP and ChBD-LGOX, lysates were used directly for adsorption under the optimal condition and the absorption effects were detected by SDS-PAGE. The absorption rate of ChBD-GFP was characterized by measuring the quality of GFP which was converted from the fluorescence intensity (Cha and Kwon, 2018) and absorption rate here was calculated as the above formula. The fluorescence intensity was detected by Hitachi F7000 fluorescence spectrophotometer. The absorption rate of ChBD-LGOX was characterized by measuring the activity of LGOX to quantitatively analyze the absorption effect of recombinant enzyme. The absorption rate was characterized by enzyme activity and was calculated as follows:

$$\text{Adsorption rate}=\left(\frac{\text{LGOX activity in absorbent}}{\text{Initial LGOX activity}}\right)\times 100\%.$$

One unit of LGOX activity was defined as the amount of enzyme that released 1 μmol H_2_O_2_ per minute. LGOX activity was measured by using a 4-aminoantipyrine method (Niu et al., 2014). The optimum catalytic conditions of different LGOX were determined under standard activity conditions using several buffers with various pH and temperature. The thermal-stability and pH-stability of free and immobilized LGOX are determined under standard activity conditions after enzyme being incubated at different conditions for 30 min, and the residual activity was determined. All binding assays above were carried out at the condition of optimal pH of chitinase *Cm*Chi1 (Zhang et al., 2018). All measurements were taken in triplicate and experiments were repeated three times to evaluate the standard deviation.

### Elution of GFP Protein From UWCP

Chitin loaded with immobilized GFP was suspended again by phosphate buffer solution (PBS) and right amount of Factor-Xa (0.2∼0.3 U/100 μg interest protein) was added. After incubation at 4°C for one night, the insoluble was separated by centrifugation while target protein was retained in the supernatant. The recovery rate was calculated as follows:

$$\text{Recovery rate}=\left(\frac{\text{GFP quality in suspension}}{\text{Initial GFP quality}}\right)\times 100\%.$$

### Production of α-KG by Transformation of L-Glutamic Acid via Immobilized LGOX

The enzymatic conversion adopted an optimized condition: 110 g/L L-glutamate, 1.5 U/mL immobilized LGOX and 250 U/mL catalase, which was used for removing H_2_O_2_ and its toxicity, in each batch of reaction (Niu et al., 2014). At the end of each batch of reaction, the product and the enzyme will be separated by centrifugation. After repeating the above steps for several times, the product concentration was determined by high performance liquid chromatography (HPLC) against a α-KG standard using a calibration curve, and then the α-KG yield from L-glutamate was calculated.

## Results

### Analysis of Putative ChBDs From *Cm*Chi1

**FIGURE 1:**
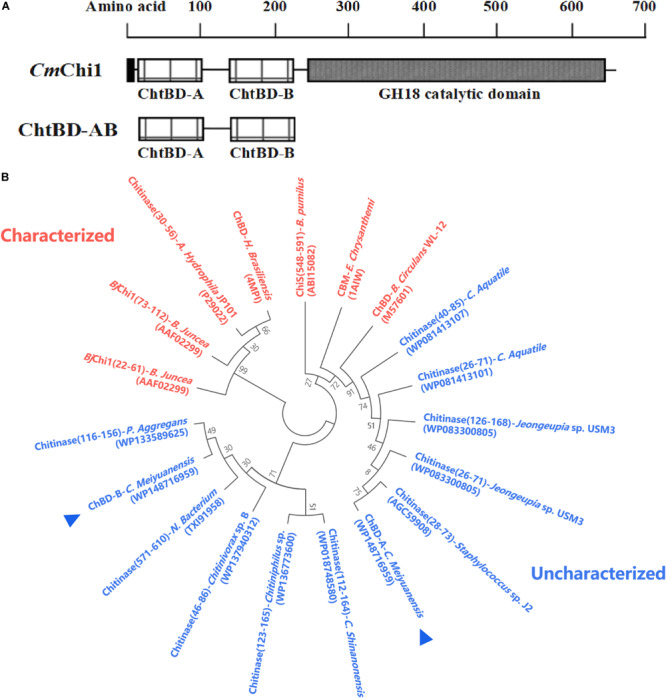
Amino acid sequence analysis of ChBD-AB. **(A)** Domain architecture of *Cm*Chi1. Domain A and B were predicted to work as chitin-binding domains which promoting the association of the enzyme with the substrate. To explore the utilization value, A and B were truncated expressed from *Cm*Chi1. **(B)** Phylogenetic analysis of chitin-binding domain A and B. Result of phylogenetic analysis indicates that both A and B has some degree of similarity with reported ChBDs and chitinases partially.

Amino acid sequence analysis revealed that *Cm*Chi1 encoded two chitin-binding modules named module A and B, respectively (Figure 1A), and both A and B belong to ChBD3 family which is widely existing in chitinases. The main structure of ChBD3 family domain is a discrete β-sandwiche and several short α-helixes between β-sandwiche providing a site for substrate binding (Vaaje-Kolstad et al., 2010). Multiple amino acid secquence alignments of the two domains revealed that ChBD-A show only 40.4% identity with ChBD of Chitinase A1 from *Bacillus circulans* WL-12 (Hashimoto et al., 2000; Ferrandon et al., 2003). The ChBD-AB show high similarity with some chitinases which have not been biochemically characterized according to phylogenetic analysis (Figure 1B). These results demonstrated that the ChBD-AB of *Cm*Chi1 are novel ChBDs which have potential value for research and application.

**FIGURE 2:**
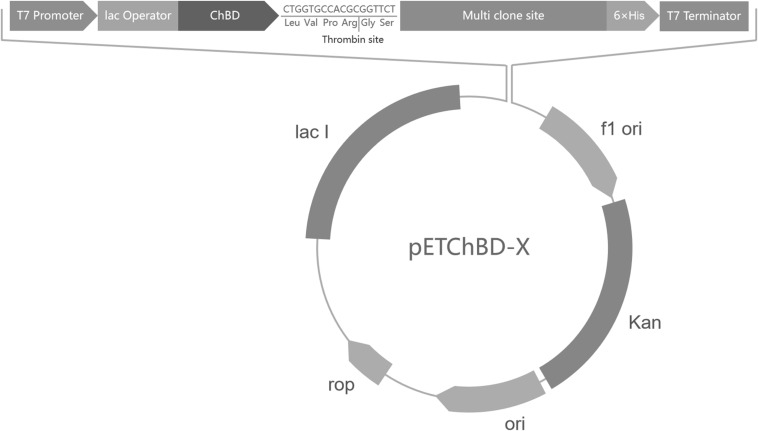
Vector plasmid with ChBD-AB as fusion tag. The common plasmid contains gene encoding ChBD as fusion tag and a Factor Xa cleavage site to separate tag and interest protein. Gene encoding interest protein can be fused at multi clone site directly.

### Parameter Optimization of Chitin-Binding Activity of ChBD-AB

The recombinant protein ChBD-AB with a C-terminal 6 × His tag was successfully expressed and purified by Ni-NTA. SDS-PAGE results showed that there were obvious specific bands same with purified proteins in the lanes with chitin matrix in chitin-binding assay (Figure 4). This phenomenon indicated that heterogenous expressed protein ChBD-AB performed great abilities of binding chitin.

**FIGURE 3:**
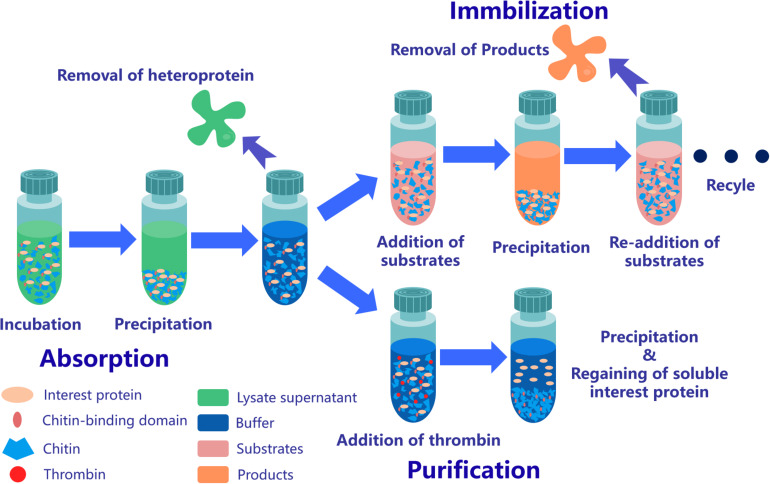
Application strategy of affinity chromatography system based on ChBD-AB. Interest protein fused ChBD can be absorbed by chitin on the purpose of separation from impurities. Obtained absorbed protein is able to be applied in repeating enzymatic conversion reaction or resolve into buffer through Factor Xa site cleavage.

**FIGURE 4:**
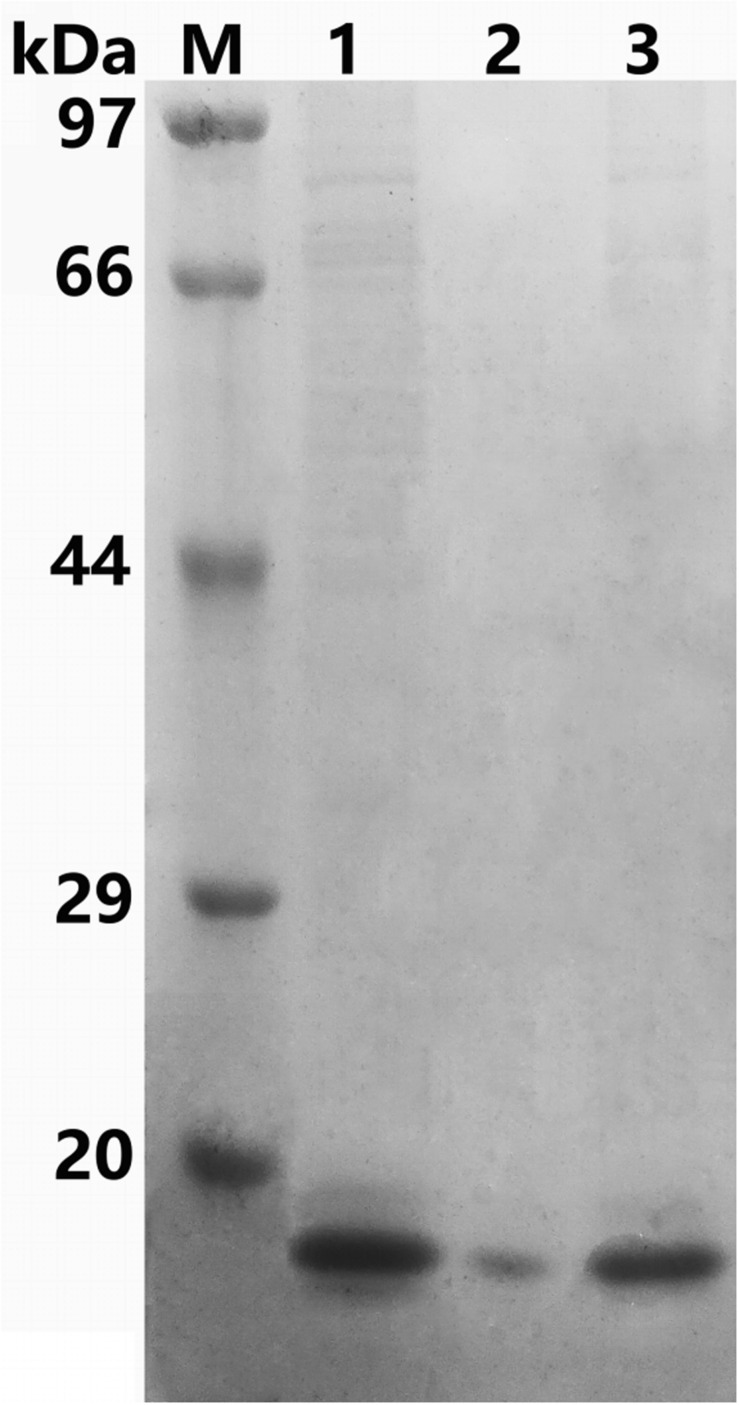
SDS-PAGE analysis of chitin-binding characterization of ChBD-AB. When compared with solution containing purified ChBD-AB (lane 1), the specific band represented ChBD-AB disappeared in the supernate after absorption (lane 2) and the protein ChBD-AB was detected in a large quantity in absorbent chitin (lane 3).

**FIGURE 5:**
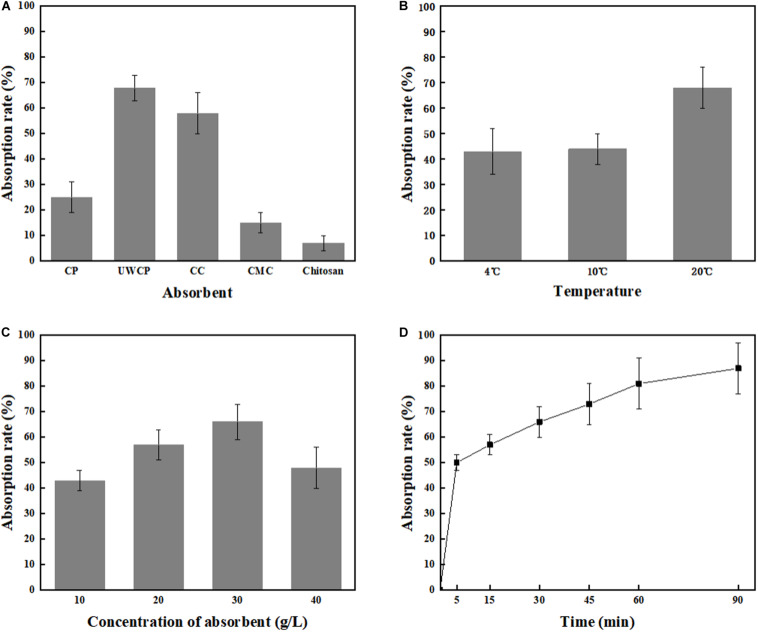
Single factor optimization of binding. **(A)** Impact of absorbent on binding. **(B)** Impact of temperature on binding. **(C)** Impact of concentration of absorbent on binding. **(D)** Impact of time on binding.

Four different factors were investigated including adsorption materials, temperature, absorbent concentration and incubating time. According to experimental data, UWCP performed best activity for binding protein ChBD-AB as the absorption rate reached 68% in 30 min while that of CC and CP were 58 and 25%, respectively (Figure 5A). For UWCP performed best in binding essay, it was used as absorbent for further optimization. The effects of temperature on adsorption efficiency was investigated. The ChBD-AB protein and UWCP were mixed and incubated at 4, 10, and 20°C respectively. For reducing the potential damage to target protein, temperature beyond 20°C is not considered in here. Absorption rate at 4 and 10°C was similar about 43%. When the temperature went up to 20°C, the adsorption rate increased significantly to 68%. Obviously, the best absorption rate was at 20°C (Figure 5B).

As shown in Figure 5C, protein ChBD-AB performed best level of absorption rate reached 66% at 30 g/L UWCP. When the concentration rised up from 10 to 30 g/L, the absorption rate rised up from 43 to 66% simultaneously. Nonetheless, the absorption rate decreased rapidly to 48% as the concentration of UWCP rised up to 40 g/L. Absorption rate was also assayed by incubation of protein ChBD-AB with UWCP for different incubation time. As shown in Figure 5D, the velocity of adsorption decreased significantly in 5 to 60 min, and the absorption rate reached 81% at 60 min. In this case, the usual experience is to choose the fastest growing stage of adsorption within 60 min.

After that, orthogonal experiments be further used for optimization of Chitin-binding activity of ChBD-AB. Experimental parameters here were set according to the results of single factor experiments. There were nine experiments to optimize binding condition. Among these experiments, the best absorption conditions were 30 g/L UWCP, at 20°C for 15 min with a highest absorption rate as 94.74% (Table 1). Further data analysis identified the optimum conditions was 30 g/L UWCP, at 20°C for 30 min when the absorption rate could reach over 95%.

**TABLE 1:** Orthogonal experiments of binding.

	Factors				Absorption rate
	Absorbent	Time	Concentration	Temperature	
1	1 (CP)	1 (15 min)	1 (20 g/L)	1 (4°C)	72.94%
2	1	2 (30 min)	2 (30 g/L)	2 (10°C)	90.47%
3	1	3 (60 min)	3 (40 g/L)	3 (20°C)	90.47%
4	2 (UWCP)	1	2	3	94.74%
5	2	2	3	1	91.04%
6	2	3	1	2	90.24%
7	3 (CC)	1	3	2	73.49%
8	3	2	1	3	80.56%
9	3	3	2	1	75.54%
I/3	84.627	80.390	81.247	79.840	
II/3	92.007	87.357	86.917	84.733	
III/3	76.530	85.417	85.000	88.590	
R^a^	15.477	6.967	5.670	8.750	

### Visible Protein Purification Process

**FIGURE 6:**
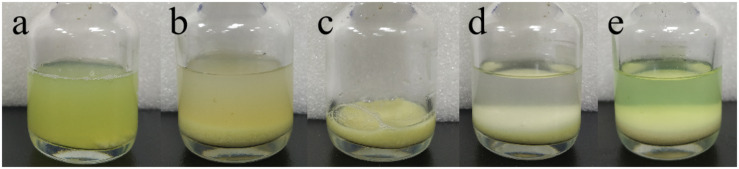
Visible purification process of GFP. **(a)** Cell lysate containing interest protein. **(b)** Cell lysate after interest protein absorbed by chitin. **(c)** Absorbent chitin with immobilized interest protein. **(d)** Buffer and absorbent chitin with immobilized interest protein before thrombin digestion. **(e)** Buffer and absorbent chitin after thrombin digestion.

**FIGURE 7:**
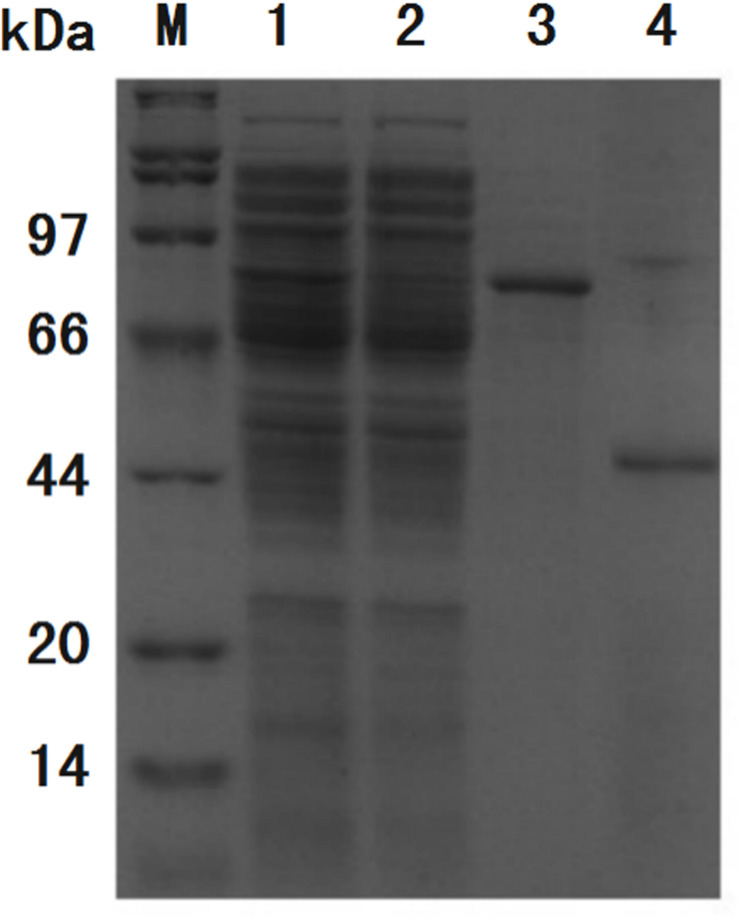
SDS-PAGE analysis of purification process of GFP. Band of interest protein ChBD-GFP disappeared in cell lysate after binding (lane 2) when compared with cell lysate before binding (lane 1), the majority of interest protein was retained in precipitate (lane 3). After thrombin digestion, GFP without ChBD could be resolved in buffer with high purity (lane 4).

After incubation of ChBD-GFP and UWCP, ChBD-GFP was recovered after Factor-Xa cleavage (Figure 3). These results demonstrated the whole purification process clearly (Figure 6). And the absorption rate was detected as 91.03% through the conversion of fluorescence intensity to quality, every gram UWCP was observed at a total loading of 3055 μg protein (Table 2). After digestion using Factor Xa, totally 75.05% of GFP without ChBD was recovered while the supernatant finally changed to clear green (Figure 6). SDS-PAGE analysis also showed the high efficiency of purification and purity of target protein (Figure 7).

**TABLE 2:** Purification process of recombinant GFP.

	Volume (mL)	Quality of interest protein (μg)	Recovery yield (%)
Lysate	30	3019.99	100
Precipitate	30	4749.21	91.03
Supernatant	30	2280.25	75.05

### One-Step Immobilization and Repeated Use of LOGX

**FIGURE 8:**
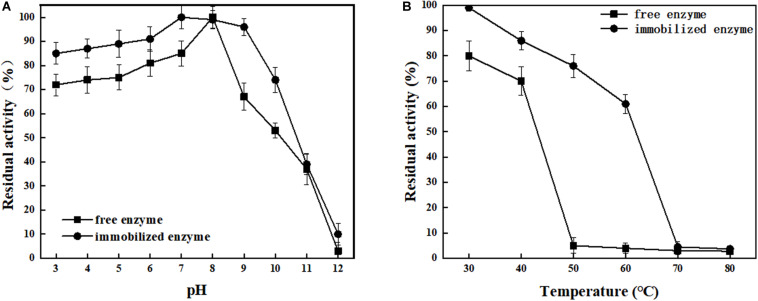
Stability analysis of immobilized and free LOGX. **(A)** pH stability. **(B)** Thermal stability. Immobilized enzyme showed much better stability than free enzyme.

ChBD-LGOX show strong ability to bind UWCP, and the process of purification and immobilization can be simplify to one step (Figure 3). According to the enzyme activity assay, high amounts of intact fusion protein are obtained with a 65% of absorption rate. Meanwhile the immobilized ChBD-LGOX showed much better thermal and pH stability than free enzyme (Figure 8).

**FIGURE 9:**
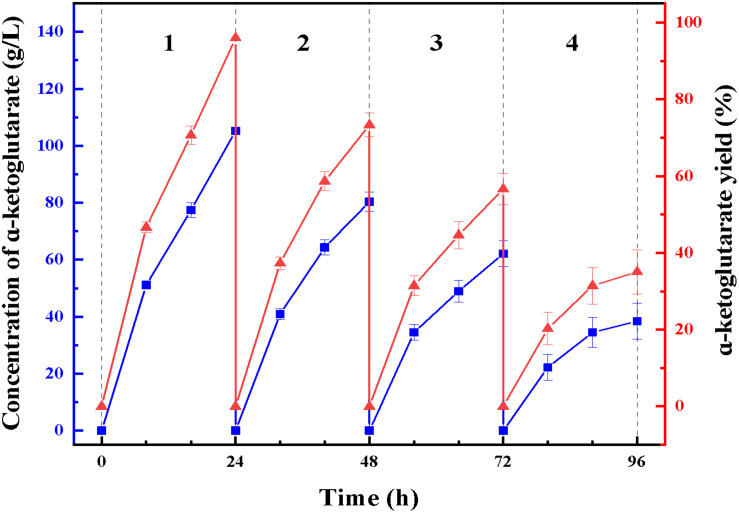
Repeating use of LOGX in enzymatic conversion of α-KG. Imobilized LGOX fused with ChBD could be reused in converting L-glutamate to α-ketoglutaric acid directly. Experiments show that this method is feasible as expected.

At enzymatic conversion assay of L-glutamate to α-ketoglutaric acid, the immobilized ChBD-LGOX showed relatively good reusability. The highest titer of -KG reached 105 g/L from 110 g/L L-glutamic acid, with conversion ratio 96% within 24 h at the first batch (Figure 9). However, the conversion rate of ChBD-LGOX decreased significantly at subsequent batches, and the conversion ratio remained of only 35% after four batches. We speculated that the yield of repeating enzymatic conversion decreased may due to the loss enzyme activity, which was consistent with the results of temperature stability (Figure 8B).

## Discussion

This paper described the construction of a universal fusion vectors pETChBD-X allowing expression of fusion proteins which can be affinity purified by a one-step procedure. The purification is based on novel ChBD-AB tag of *Cm*Chi1 which under physiological conditions binds to cheap chitin waste. After parameter optimization, the optimal binding condition was 30 g/L UWCP and incubating at 20°C for 30 min. These conditions are very advantageous for recombinant protein purification. Firstly, chitin is a kind of absolutely non-polluting material as it is natural. Secondly, the time, cooling and reagent costs will be further compressed by optimization. Generally speaking, many reported cases of protein purification based on carbohydrate-binding domain usually need overnight incubating such as Myung et al. (2011) immobilized phosphoglucose isomerase through cellulose-binding module-tagged thermophilic enzyme which took a lot of time to incubate. The operation at 20°C was near room temperature and operation process can be greatly simplified with adsorption and centrifugation. For example, Ramirez et al. (2013) used a CBM as fusion tag to immobilize endo-b-*N*-acetylglucosaminidase and the system should be incubated at 4°C which increased thermal insulation costs greatly. What’s more, the price of chitin is very low and the preparing process of UWCP is relatively simple as well. Thirdly, the whole process of purification or immobilization is simple, only incubation and centrifugation are needed.

By all accounts, the protein purification and immobilization method provided in this study is suitable for large-scale industrial application because of its unique advantages. Recombinant protein ChBD-GFP also showed good bonding property, 91% interest was absorbed under the same condition and 75% was recovered after factor-Xa digestion. The visualization process intuitively reflects these steps and SDS-AGE analysis confirmed the high-purity of interest protein. Recovery yield data is much higher than reported cases, for instance, Leister applied chitin-binding domain in immobilizing β-galactosidases and received activity retention of 55% (Leister, 2014); Ramirez et al. (2013) immobilized pectinase on an alginate-coated chitin support by adsorption and the yield of immobilized protein was 70% and the enzyme retained 60% of the initial activity.

Another recombinant protein ChBD-LGOX was successfully purified and immobilized in single step which revealed the convenience of this method. High amounts of intact fusion protein are produced which can be immobilized on UWCP in a yield (65%). Moreover, the stability of enzyme was significantly enhanced after immobilization. This is consistent with some reported cases, for example, the *cis*-epoxysuccinate hydrolases fused with cellulose-binding domain and immobilized on cellulose showed better stability than its wild-type version (Cui et al., 2012); Chern and Chao (2005) immobilized D-allantoinase by using chitin-binding domain from *Bacillus circulans* WL-12, the active half-life of immobilized enzyme was greatly prolonged. The characterization of ChBD-LGOX changed especially the enzymatic activity decreased by a big margin which revealed an obvious disadvantage of this immobilization approach. Stereospecific blockade between fusion tag and target protein might be the main reason for this phenomenon, further research is needed to overcome this shortage. When applied in enzymatic conversion, the immobilized enzyme also showed good value, average conversion rate kept above 65% even within four batches of conversion reaction, averagely 72 g/L α-ketoglutarate could be produced from 110 g/L L-glutamate. Though the yield was relatively low when compared with reported cases, such as Ödman et al. (2004) Used a coupled system L-glutamate dehydrogenase/NADH oxidase to convert nearly 100% L-glutamateto to α-ketoglutarate, the most significant advantage of the enzymatic conversion method in this study is that immobilized enzyme could be used repeatedly. Although the yield of repeating enzymatic conversion decreased by a big margin, this is mainly due to the relatively poor stability of the enzyme itself. Thus, the recombinant enzyme ChBD-LGOX may after all be accepted as an appropriate tool for producing α-ketoglutarate with energy saving and low consumption.

## Conclusion

In summary, the ChBD-AB affinity tag system described here provides a novel tool for an innovative alternative method for purifying, immobilizing, and spreading fusion proteins. Great advantages including low cost, no pollution, easy operation, rapidness, and high-purity suggested that the plasmid vector pETChBD-X has potential value in immobilization and purification of enzymes.

## Data Availability Statement

The raw data supporting the conclusions of this article will be made available by the authors, without undue reservation.

## Author Contributions

JZ was responsible for experimental design and thesis writing. JC and NZ were responsible for experimental operation. AZ and KC provided experimental materials. NX, FX, WZ, MJ, and WD contributed to data analysis and polishing thesis.

## Conflict of Interest

The authors declare that the research was conducted in the absence of any commercial or financial relationships that could be construed as a potential conflict of interest.
